# Supplementary material for: Innovative Integration of 4D Cardiovascular Reconstruction and Hologram: Framework Development of a New Visualization Tool for Coronary Artery Bypass Grafting Planning
Source: JMIR Med Inform. 2025 Sep 30;13:e72237. doi: 10.2196/72237 (PMC12483336; doi:10.2196/72237)
Supplement: Multimedia Appendix 1 [file medinform-v13-e72237-s001.doc]

**Holographic Experience Reviews**

1. **It creates more awareness of spatial orientation of anatomical structures.**

| **5**  **Strongly Agree** | **4**  **Agree** | **3**  **Neither Or N/A** | **2**  **Disagree** | **1**  **Strongly Disagree** |
| --- | --- | --- | --- | --- |

1. Identification of coronary calcified plaque is easy

| **5**  **Strongly Agree** | **4**  **Agree** | **3**  **Neither Or N/A** | **2**  **Disagree** | **1**  **Strongly Disagree** |
| --- | --- | --- | --- | --- |

1. **Perception of coronary travel depth trends is clear**

| **5**  **Strongly Agree** | **4**  **Agree** | **3**  **Neither Or N/A** | **2**  **Disagree** | **1**  **Strongly Disagree** |
| --- | --- | --- | --- | --- |

1. Coronary artery and heart dynamic imaging is realistic

| **5**  **Strongly Agree** | **4**  **Agree** | **3**  **Neither Or N/A** | **2**  **Disagree** | **1**  **Strongly Disagree** |
| --- | --- | --- | --- | --- |

1. Can clearly identify pericardial adhesions

| **5**  **Strongly Agree** | **4**  **Agree** | **3**  **Neither Or N/A** | **2**  **Disagree** | **1**  **Strongly Disagree** |
| --- | --- | --- | --- | --- |

1. Rotate, zoom and other tools are easy to use

| **5**  **Strongly Agree** | **4**  **Agree** | **3**  **Neither Or N/A** | **2**  **Disagree** | **1**  **Strongly Disagree** |
| --- | --- | --- | --- | --- |

1. In obtaining the information needed for preoperative evaluation, it provides more intuitive and comprehensive insights compared to observing static three-dimensional cardiovascular CT images on a two-dimensional screen.

| **5**  **Strongly Agree** | **4**  **Agree** | **3**  **Neither Or N/A** | **2**  **Disagree** | **1**  **Strongly Disagree** |
| --- | --- | --- | --- | --- |

**Overall evaluation of the hologram system experience：**

| **5**  **Excellent** | **4**  **Good** | **3**  **Fair** | **2**  **Poor** | **1**  **Very poor** |
| --- | --- | --- | --- | --- |

**Attitude towards (future) use of Holographic Imaging System**

1. It is helpful for preoperative planning of coronary artery bypass surgery

| **5**  **Strongly Agree** | **4**  **Agree** | **3**  **Neither Or N/A** | **2**  **Disagree** | **1**  **Strongly Disagree** |
| --- | --- | --- | --- | --- |

1. It is beneficial for clinical demonstration and teaching

| **5**  **Strongly Agree** | **4**  **Agree** | **3**  **Neither Or N/A** | **2**  **Disagree** | **1**  **Strongly Disagree** |
| --- | --- | --- | --- | --- |

1. It is useful for preoperative patient education

| **5**  **Strongly Agree** | **4**  **Agree** | **3**  **Neither Or N/A** | **2**  **Disagree** | **1**  **Strongly Disagree** |
| --- | --- | --- | --- | --- |

1. There is a desire for routine use of the system

| **5**  **Strongly Agree** | **4**  **Agree** | **3**  **Neither Or N/A** | **2**  **Disagree** | **1**  **Strongly Disagree** |
| --- | --- | --- | --- | --- |
